# Estimating the Under-ascertainment of COVID-19 cases in Toronto, Ontario, March to May 2020

**DOI:** 10.1177/22799036231174133

**Published:** 2023-05-12

**Authors:** Binyam N Desta, Sylvia Ota, Effie Gournis, Sara M Pires, Amy L Greer, Warren Dodd, Shannon E Majowicz

**Affiliations:** 1 School of Public Health Sciences, University of Waterloo, Waterloo, ON, Canada; 2 Toronto Public Health, Toronto, ON, Canada; 3 Risk-Benefit Research Group, Technical University of Denmark, Lyngby, Denmark; 4 Department of Population Medicine, University of Guelph, Guelph, ON, Canada

**Keywords:** COVID-19, surveillance, disease, disease notification, report, public reporting of healthcare data, Ontario, Canada

## Abstract

**Background:**

Public health surveillance data do not always capture all cases, due in part to test availability and health care seeking behaviour. Our study aimed to estimate under-ascertainment multipliers for each step in the reporting chain for COVID-19 in Toronto, Canada.

**Design and methods:**

We applied stochastic modeling to estimate these proportions for the period from March 2020 (the beginning of the pandemic) through to May 23, 2020, and for three distinct windows with different laboratory testing criteria within this period.

**Results:**

For each laboratory-confirmed symptomatic case reported to Toronto Public Health during the entire period, the estimated number of COVID-19 infections in the community was 18 (5th and 95th percentile: 12, 29). The factor most associated with under-reporting was the proportion of those who sought care that received a test.

**Conclusions:**

Public health officials should use improved estimates to better understand the burden of COVID-19 and other similar infections.

## Background

Since its first case of COVID-19 on January 23, 2020,^1^ Canada has experienced at least four epidemic waves to date, with reported cases surpassing 2.1 million as of December 30, 2021.^2^ More than one-third of these cases occurred in the province of Ontario, of which about one-third occurred in Ontario’s largest city, Toronto.^2,3^ Given the factors and chain of steps in disease reporting, these case numbers are likely under reported,^4–7^ for example, because individuals do not seek care for their symptoms, or due to changes in testing criteria.^6–8^

COVID-19, a new disease introduced into a fully susceptible global population, will likely continue to impact population health going forward. To better understand the impact of the pandemic, several studies have estimated the number of individuals who became infected with COVID-19 in the community.^6,7,9–20^ Serological surveys, that test for any previous infection with COVID-19, are the most common method used to estimate the number of individuals infected with COVID-19 and are useful when there are asymptomatic cases.^9–13,15,17–20^ However, because serological surveys often use small or un-representative population samples, complementing serosurvey findings with estimates of the fraction of cases reported at other steps in the disease reporting chain may allow more valid estimation.^21^

This approach to assessing under-ascertainment of notifiable communicable diseases by estimating the fraction of cases captured at each step in the reporting chain has been used for foodborne infections and influenza.^22–24^ Here, we applied this method to COVID-19 cases reported in Toronto, Ontario, Canada, during the first wave of the pandemic, when lab testing was limited to those meeting specific criteria (March to May 2020). Our objectives were to estimate multipliers for each step in the reporting chain for COVID-19, and overall, to better understand the extent of COVID-19 infection in the general community in Toronto during the early stages of the pandemic. This methodology may be useful for other stages of the pandemic, such as the wave that began in Ontario in December 2021, driven by the Omicron variant, where demand for laboratory-based PCR testing rapidly exceeded capacity limits, and testing eligibility became restricted primarily to those in high-risk settings.^25^

## Methods

### Approach

**Figure 1.**
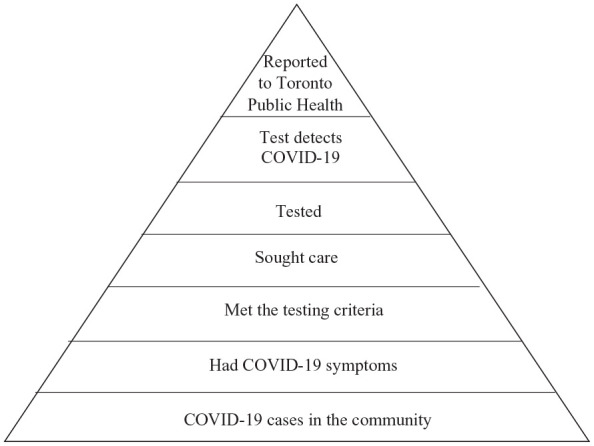
Reporting pyramid for COVID-19 in Toronto, Ontario, showing the sequential steps necessary for case capture in the local public health surveillance system, March to May 2020.

COVID-19 cases living in Toronto (outside long-term care facilities) with onset dates any time from the beginning of the pandemic through to May 23, 2020, were included. We chose this end date because lab testing became widely available to anyone with symptoms on May 24, 2020. Given Ontario’s testing criteria changed as the pandemic unfolded, we estimated under-ascertainment both overall and for three different testing criteria windows within this period. We used information from three existing datasets to estimate the proportion of cases captured at major steps within the communicable disease reporting chain (Figure 1).

Because we expected uncertainty in these estimates, we specified proportions using input distributions instead of discrete values (Table 1). We multiplied distributions using a stochastic model, taking the inverse to obtain the overall and testing window-specific under-ascertainment estimates.

**Table 1.** Input distributions for, and mean estimates of, the proportions reported at each step in the reporting chain, in the model to estimate under-ascertainment rate of COVID-19 cases in Toronto, Ontario, Canada, March to May 2020.

Reporting chain step (Data source)	Time							
	Entire timeframe		Testing criteria window					
			Up to March 12, 2020		March 13–April 9, 2020		April 10–May 23, 2020	
	Input distribution (parameters)*	Mean (5^th^, 50th, 95th)	Input distribution (parameters)	Mean (5^th^, 50th, 95th)	Input distribution (parameters)	Mean (5^th^, 50th, 95th)	Input distribution (parameters)	Mean (5^th^, 50th, 95th)
Proportion of those who test positive who were reported to Toronto Public Health (**, ***)	Beta (5530 + 1, 6929–5530 + 1)	0.798(0.790, 0.798, 0.806)	Beta (57 + 1, 87 – 57 + 1)	0.651 (0.566, 0.653, 0.732)	Beta (1341 + 1, 1709 − 1341 + 1)	0.784 (0.768, 0.784, 0.801)	Beta (4132 + 1, 5133 − 4132 + 1)	0.805 (0.796, 0.805, 0.814)
Proportion of people with COVID-19 who tested positive-test sensitivity (****)	Uniform (0.707, 0.880)	0.794 (0.716, 0.796, 0.872)	Uniform (0.707, 0.880)	0.794 (0.716, 0.796, 0.872)	Uniform (0.707, 0.880)	0.794 (0.716, 0.796, 0.872)	Uniform (0.707, 0.880)	0.794 (0.716, 0.796, 0.872)
Proportion of those who sought care that got tested (*****)	Pert (0.17, 0.75, 1.00)	0.695 (0.425, 0.708, 0.920)	Pert (0.18, 0.75, 1.00)	0.699 (0.434, 0.710, 0.922)	Pert (0.16, 0.75, 1.00)	0.689 (0.417, 0.702, 0.914)	Pert (0.22, 0.75, 1.00)	0.703 (0.446, 0.713, 0.918)
Proportion of those who met the testing criteria who sought care (*****)	Beta (168 + 1, 461 – 168 + 1)	0.365 (0.328, 0.364, 0.403)	Beta (34 + 1, 87 – 34 + 1)	0.394 (0.310, 0.393, 0.478)	Beta (131 + 1, 331 – 131 + 1)	0.397 (0.353, 0.396, 0.441)	Beta (46 + 1, 128 – 46 + 1)	0.362 (0.294, 0.362, 0.433)
Proportion of those with COVID symptoms who met the testing criteria (*****)	Beta 461 + 1, 1062 – 461 + 1)	0.434 (0.409, 0.434, 0.459)	Beta (87 + 1, 262 – 87 + 1)	0.330 (0.284, 0.329, 0.378)	Beta (331 + 1, 871 – 331 + 1)	0.381 (0.354, 0.380, 0.408)	Beta (128 + 1, 224 – 128 + 1)	0.570 (0.516, 0.571, 0.625)
Proportion of those with COVID-19 in the community who were symptomatic (**)	Beta (8091 + 1, 9701 − 8091 + 1)	0.834 (0.828, 0.834, 0.840)	Beta (8091 + 1, 9701 − 8091 + 1)	0.834 (0.828, 0.834, 0.840)	Beta (8091 + 1, 9701 − 8091 + 1)	0.834 (0.828, 0.834, 0.840)	Beta (8091 + 1, 9701 − 8091 + 1)	0.834 (0.828, 0.834, 0.840)

* The types of distribution (parameters): Beta (numerator + 1, denominator-numerator + 1); Uniform (minimum, maximum); Pert (minimum, most likely value, maximum).

** Toronto Public Health notifiable disease surveillance data, January–August, 2020.

*** Weekly count data of COVID-19 diagnostic test recipients and positives, January–September, 2020.

**** Published studies.^26–29^

***** COVID-19 population survey conducted by Toronto Public Health, April–May, 2020.

We also calculated step-specific under-ascertainment estimates by multiplying proportions at higher steps in the reporting chain and taking the inverse. Assumptions and analytic decisions were explored in a sensitivity analysis (Supplemental Table S1) and final under-ascertainment estimates were compared to those from serosurvey data for the same timeframe and population.^30^ We used a seroprevalence study from Public Health Ontario^30^ to determine the ratio of the estimated number of cases in the total population to the number of cases reported to TPH and compared the resulting ratios to our model-based estimates.

### Ethical clearance

This study received ethical clearance from a University of Waterloo Research Ethics Committee (#42591).

### Time windows

The first time window was up to and including March 12, 2020, when testing was offered to only those with symptoms consistent with COVID who also had: traveled to an impacted area; close contact with a confirmed or probable COVID case; or close contact with a person with acute respiratory illness who had been to an impacted area. Health providers could request testing outside of these parameters based on assessment and clinical judgment.^31^

The second window was between March 13 and April 9, 2020, where priority testing was done for people who: had severe symptoms and thus required hospitalization; were health care workers or who work in a health care setting; were first responders (police officer, firefighter, paramedic, correctional officer, parole officer, or probation officer); were at high risk (i.e. people with lung or heart disease, diabetes or health conditions that affect their immune system); or were living in congregate living facilities. Travel history was no longer a criterion for testing. Mildly symptomatic individuals were asked to self-isolate at home and were not prioritized for testing.^31^

The third window was between April 10 and May 23, 2020, where the criteria from the second window were extended to include people who were: essential workers; residents/staff of homeless shelters or group homes; or living with health care workers.^32^ In this window, there was an additional criterion announced on April 15, 2020, which added enhanced/surveillance testing of all residents and staff in long-term care homes^33^; however because our data did not include information for these people specifically, this criterion was ignored in this analysis.

### Data sources

We used three datasets: Toronto’s notifiable disease surveillance data on reported cases of COVID-19 (de-identified) from January to May 2020 (“reported case data”); weekly counts of the number of COVID-19 tests completed and numbers testing positive in Toronto from January to May 2020 (“weekly testing data”); and a survey of the general population conducted by Toronto Public Health in April–May 2020 (“population survey data”).

The reported case data included information on case classification (confirmed, probable), gender (male, female, transgender, other, unknown), symptomatic status (symptomatic, asymptomatic, unknown), age, specimen collection dates (range: January 23, 2020–May 23, 2020), and reported dates (range: January 23, 2020–May 23, 2020). Of the 9014 cases of COVID-19 reported in the general community, 6217 (72.2%) were symptomatic, of which 5530 (90.1%) were laboratory-confirmed, and were thus included in our analysis. We excluded probable cases that did not have laboratory confirmation, as a criterion for being defined as a probable case required symptoms which would increase the proportion symptomatic. For the 5530 cases, all variables were 100% complete except the specimen collection date, which was missing for 24 (0.43%) cases.

Weekly COVID-19 testing data from the Ontario Laboratory Information System were received from the Institute of Clinical Evaluative Sciences.^34^ They included weekly tallies of the number of people with a positive test result outside of long-term care settings. For individuals with more than one confirmed positive COVID-19 test, only the first testing episode (specimen collected, test positive) was included in the weekly counts. We included 19 complete weekly counts in our analysis (from January 12 to May 23, 2020), comprising 6929 people testing positive. Because any numbers less than six were suppressed to protect privacy, there were two weeks (January 19–25, 2020, and February 16–22, 2020) where the numbers of people who tested positive were suppressed.

The population survey was administered through Toronto Public Health’s public-facing website^35^ in April–May 2020, using CheckMarket survey software (Checkmarket^®^, Turnhout, Belgium^36^). The survey was publicized via social media and collected information on COVID-19 symptoms between March 1 and May 25, 2020. For Toronto residents reporting symptoms, the survey then asked about: age; gender (male, female, transgender, “other”); symptoms; the dates symptoms began and resolved; and possible exposures (i.e. travel history outside Canada within 14 days of symptoms onset, any contact with either a confirmed COVID-19 case or an individual with COVID-19 symptoms who was not tested for COVID-19, health care worker or worker in the health care setting, first responder, worked with homeless clients, provided essential services, or interacted with the public). The survey also asked about: testing for COVID-19; care seeking (visiting an emergency department or an assessment center; contacting Telehealth, Toronto Public Health, or their family physician); and chronic or other underlying medical conditions.

A total of 3532 (~0.12% of the Toronto population) people completed the survey between April 2 and May 25, 2020, of whom 3529 (99.9%) reported living in Toronto. Of these, 2302 (65.2%) provided their symptomatic status, of whom 1444 (62.7%) reported having symptoms of COVID-19. Data completeness by variables is given in Supplemental Table S2. After data manipulation (see Supplemental Materials, “Additional Notes on Data Sources”), the remaining 1433 symptomatic respondents had onset dates from September 27, 2019, to May 30, 2020, and resolved dates from January 2 to June 5, 2020. Of the 1433 symptomatic respondents, 360 (25.1%) reported neither onset nor resolved dates, and 11 (0.8%) reported impossible dates (*n* = 4, onset or resolved date after the survey date; *n* = 7, onset date after resolved date). We excluded these 371 people from our main analysis.

### Analysis

We used SAS 9.4 (SAS Institute, Cary, NC) to perform data manipulation and calculate parameters for input distributions (Table 1), as described below. We generated under-ascertainment estimates using Monte Carlo simulation modelling with 10,000 iterations in R version 4.0.2. For the time window-specific analyses, data were matched to time windows (Supplemental Table S3). Population survey respondents and reported COVID-19 cases who were missing data for specific variables were excluded from the analysis of that variable. We treated suppressed weekly count numbers as zeros in the main analyses.

#### Proportion of those who test positive who were reported to Toronto Public Health

The input distribution for the proportion of those who tested positive who were reported to Toronto Public Health was determined using the weekly count data and reported case data. We parameterized a beta distribution where the denominator was the number of positive tests within the testing window. Two of these counts spanned two testing windows; for these, we assumed numbers were evenly distributed across the seven days. We divided these numbers between testing windows proportional to the number of days per testing window. For example, testing week March 8–14 spanned testing windows one (5 days) and two (2 days); thus 5/7 of the number of positives were assigned to testing window one and 2/7 to testing window two. The numerator was the number of COVID-19 cases reported during the testing window. This was determined using the reported dates in the reported case data (Supplemental Table S3). For the overall estimate, the denominator and numerator were the total number of positive tests and total number of COVID-19 cases reported, respectively.

#### Proportion of those who were tested that tested positive

The input distribution for the proportion of those who got tested that tested positive was estimated by applying COVID-19 test sensitivity. The test in use to detect COVID-19 in Ontario, Canada, was polymerase chain reaction (PCR) with varieties including real-time (qPCR), real-time reverse-transcriptase (rRT-PCR), and reverse transcriptase (RT-PCR).^37^ We used reported test sensitivities for these tests: qPCR of 71%^27^; rRT-PCR range from 70.7% to 83.3%^28,29^; and RT-PCR range from 71% to 88%.^26^ We parameterized a uniform distribution with minimum (70.7%) and maximum (88%) test sensitivity values.

#### Proportion of those who sought care that got tested

The input distribution for the proportion of those who sought care that got tested was determined using information from several sources. We started with the population survey, where the denominator was the number of individuals symptomatic during the testing window who reported seeking medical care, estimated as those who reported that they: went to an emergency department or an assessment center; called Telehealth (i.e. Ontario’s free telephone service to get health advice or information from a Registered Nurse); contacted Toronto Public Health; or contacted their family physician. The numerator was the subset who were tested, as reported in the survey. For the overall estimate, the denominator and numerator were the total number of respondents (counting only those who met the testing criteria at least once in any of the windows) who reported seeking care and getting tested, respectively, regardless of whether they were able to be matched to a testing window. However, in the context of the global pandemic, the estimates (range: 0.17–0.23) look too small. Thus, unlike the underreporting estimation of foodborne illnesses, we decided to use these numbers as minimum values in pert distribution and the maximum value to be one (100%). The most likely value we used was the highest proportion (i.e. 75%) during a pandemic, as reported by Reed et al. ’s paper on the influenza A pandemic (H1N1),^38^ for the proportion of persons seeking care with a specimen collected.

#### Proportion of those who met the testing criteria who sought care

The input distribution for the proportion of individuals who met the testing criteria who sought care was determined using population survey data. We parameterized a beta distribution, where the denominator was the number of symptomatic individuals within the testing window who met the testing criteria. The numerator was the subset who sought medical care. For the overall estimate, the denominator and numerator were the sum (counting only those who met the testing criteria at least once in any of the windows) of the testing window-specific denominators and numerators, respectively.

#### Proportion of those with symptoms who met the testing criteria

The input distribution for the proportion of those with COVID-19 symptoms who met the testing criteria was determined using information for symptomatic individuals from the population survey. We parameterized a beta distribution, where the denominator was the number of individuals symptomatic during the testing window. This was determined using reported onset and resolved dates (Supplemental Table S3) where individuals were included in the testing window if either (a) their onset and resolved dates encompassed some or all of the testing window (meaning some individuals could contribute data to more than one testing window), or (b) for those with only one date, said date fell within the window. The numerator was the subset of individuals with COVID-19 symptoms who met the testing criteria for the window. Finally, for the overall estimate, the denominator was all those who were symptomatic (regardless of whether they were able to be matched to a testing window), and the numerator was the number of people who met the testing criteria at least once in any of the windows.

#### Proportion of those with COVID-19 in the community who were symptomatic

The input distribution for the proportion of those with COVID-19 in the community who were symptomatic was determined using the proportion symptomatic in the reported case data; we prioritized using data from the study population and timeframe, since values from the literature from other similar settings reported highly varying asymptomatic proportions (ranges: 20%–76%; Supplemental Table S4). We parameterized a beta distribution, where the denominator was the number of confirmed COVID-19 cases reported to Toronto Public Health between January 1 and May 23, 2020. The numerator was number of these cases who were symptomatic.

## Results

The cumulative numbers of illnesses at each step in the reporting chain are given overall and by testing window (Table 2).

**Table 2.** Estimated cumulative number of COVID-19 cases captured at each step in the reporting chain and occurring in the community for each case of COVID-19 reported to Toronto public health, Ontario, Canada, March to May 2020.

Reporting chain step	Mean (fifth, Median, 95th)			
	Entire timeframe	Testing criteria window		
		Up to March 12, 2020	March 13–April 9, 2020	April 10–May 23, 2020
Reported to Toronto Public Health	1 (1, 1, 1)	1 (1, 1, 1)	1 (1, 1, 1)	1 (1, 1, 1)
Test detects COVID-19	1.253 (1.241, 1.253, 1.266)	1.545 (1.367, 1.531, 1.766)	1.275 (1.249, 1.275, 1.303)	1.243 (1.229, 1.242, 1.257)
Tested	1.584 (1.438, 1.577, 1.752)	1.953 (1.656, 1.935, 2.313)	1.612 (1.460, 1.604, 1.783)	1.570 (1.425, 1.562, 1.738)
Sought care	2.419 (1.678, 2.240, 3.782)	2.954 (1.995, 2.745, 4.598)	2.485 (1.719, 2.290, 3.861)	2.348 (1.672, 2.193, 3.547)
Met the testing criteria	6.652 (4.520, 6.159, 10.425)	7.636 (4.796, 7.102, 12.317)	6.289 (4.241, 5.804, 9.936)	6.580 (4.370, 6.175, 10.290)
Had COVID-19 symptoms	15.340 (10.337, 14.208, 24.083)	23.351 (14.100, 21.634, 38.072)	16.551 (11.080, 15.258, 26.140)	11.574 (7.566, 10.859, 18.087)
COVID-19 cases in the community	18.395 (12.390, 17.044, 28.897)	28.001 (16.910, 25.963, 45.557)	19.846 (13.292, 18.295, 31.307)	13.879 (9.074, 13.019, 21.661)

**Figure 2.**
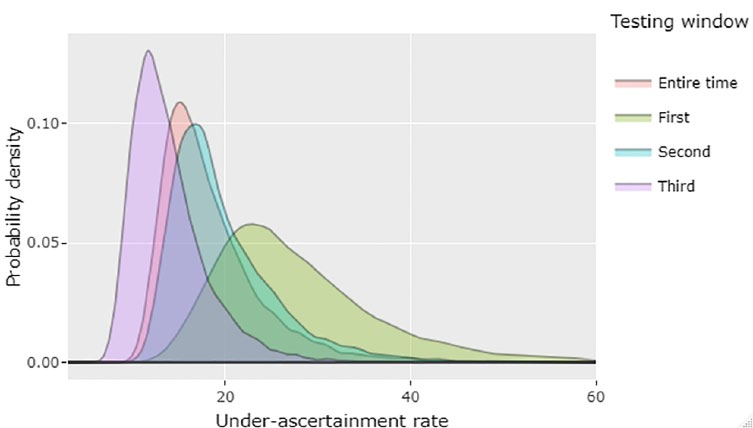
Distribution of the estimated overall under-ascertainment rate (for the different windows and entire period) of COVID-19 cases in Toronto, Ontario, showing the number of COVID-19 illness in the community for each case reported to Toronto Public Health, Ontario, Canada, March–May 2020.

The density distribution curves for the under-ascertainment estimates (Figure 2) show that the under-ascertainment improved across the three testing windows, and uncertainty about the multiplier estimates decreased.

For the overall window, we estimated a median of 17 and a mean of 18 individuals with COVID-19 in the community for each symptomatic, laboratory-confirmed COVID-19 case reported to Toronto Public Health. Thus, the 5530 symptomatic, laboratory-confirmed cases reported to Toronto Public Health represented an estimated ~ 99,540 (5th and 95th percentile: 66,360, 160,370) community cases of COVID-19 from March to May 2020. Uncertainty about the proportion of those who sought care that received a test made the highest contribution to the uncertainty of the overall under-ascertainment rate (Table 3).

**Table 3.** Correlation between the distribution of the overall under-ascertainment multiplier and the input distributions for the step-specific proportion in the reporting chain (for the entire period only and presented in descending order of correlation), Toronto Public Health, Ontario, Canada, March to May 2020.

Proportion	Correlation coefficient (Pearson’s)	Significance (α = 0.05)
Tested	0.913	<0.001
Sought care	0.259	<0.001
Test detects COVID-19	0.258	<0.001
Met the testing criteria	0.152	<0.001
Reported to Toronto Public Health	0.038	<0.001
Had COVID-19 symptoms	0.036	<0.001

Although our estimated under-ascertainment multiplier (18) was higher than that from the COVID-19 seroprevalence study for Ontario during the same timeframe (11; Table 4), our estimate and its uncertainty interval (18; 12, 29) fell within the estimated range of values when using seroprevalence data (2, 47).

**Table 4.** COVID-19 seroprevalence, number of cases in the total population, and infection under-ascertainment ratio of reported cases to estimated number of cases in Toronto, Ontario, Canada, by period, March to May 2020.

Age and Sex Category		Seroprevalence estimates by Public Health Ontario (95% CI)^30^	Total population in Toronto^34^	(A) Estimated number of cases in the total population using seroprevalence estimates (95% CI)	(B) Number of cases reported to TPH	Ratio of (A) to (B) (95% CI)
March–April 2020						
0–19 years	Male	0.0 (0.0, 4.5)	293,536	0 (0, 13209)	28	UTD* (UTD, 471.75)
	Female	0.0 (0.0, 4.3)	278,027	0 (0, 11955)	29	UTD (UTD, 412.24)
20–59 years	Male	0.8 (0.02, 4.2)	872,532	6980 (175, 36646)	1331	5.24 (UTD, 27.53)
	Female	0.0 (0.0, 1.2)	900,041	0 (0, 10801)	1125	UTD (UTD, 9.60)
≥60 years	Male	2.8 (0.3, 9.7)	324,386	9083 (973, 31465)	490	18.54 (1.99, 64.21)
	Female	0.0 (0.0, 6.3)	387,204	0 (0, 24394)	410	UTD (UTD, 59.50)
Total			3, 055, 726	16063 (1148, 128470)	3413	4.71 (UTD, 37.64)
May 2020						
0–19 years	Male	0.0 (0.0, 4.4)	293,536	0 (0, 12916)	43	UTD (UTD, 300.37)
	Female	1.4 (0.2, 4.8)	278,027	3892 (556, 13345)	64	60.81 (8.69, 208.52)
20–59 years	Male	1.6 (0.4, 4.1)	872,532	13961 (3490, 35774)	760	18.37 (4.59, 47.07)
	Female	2.6 (0.6, 4.7)	900,041	23401 (5400, 42302)	724	32.32 (7.46, 58.43)
≥60 years	Male	1.1 (0.1, 4.0)	324,386	3568 (324, 12975)	258	13.83 (1.26, 50.29)
	Female	0.5 (0.01, 2.9)	387,204	1936 (39, 11229)	217	8.92 (UTD, 51.75)
Total			3, 055, 726	46758 (9809, 128541)	2066	22.63 (4.75, 62.22)
Overall (March to May 2020)						
			3, 055, 726	62821 (10957, 257011)	5479**	11.47 (2.00, 46.91)

* UTD—Unable to determine because the denominator was lower than the numerator.

** 5479 was smaller than the total cases (5530) used in the model because there were people with other gender categories than male/female in the reported case data, but the serosurvey reported in male/female categories only.

## Discussion

Our study estimated under-ascertainment multipliers for each step in the reporting chain for COVID-19 in Toronto, Canada, from March to May 2020. Our analysis suggests that tens of thousands of COVID-19 infections occurred in the community during the earlier phase of the pandemic, before lab testing became widely available, and that approximately 5.6% of COVID-19 infections were captured through routine public health disease surveillance during this early pandemic phase (March–May 2020). A nationwide study in the US that used similar methods estimated that 13% of community cases were captured through PH disease surveillance, although their study period extended until the end of September 2020.^14,39^ This lower estimated under-reporting in the US study may reflect improved case ascertainment as the pandemic unfolded, as both the US study and ours demonstrated decreased uncertainty in the under-ascertainment multiplier, and improved case capture, across study periods. The difference may also be due to differences in test sensitivity particularly given the US study’s longer duration or potentially more COVID-19 cases in the US, and differences in laboratory access, health-seeking behaviour, data sources, test eligibility, and other factors. Additionally, factors related to health-seeking behaviour may have impacted reporting of symptoms, including the belief that an illness was not COVID-19 (since COVID-19 symptoms are relatively non-specific), perceived limited benefit to reporting (as there was no treatment available at the time nor was reporting mandatory), stigma related to having COVID-19, and avoiding the health care system when mildly ill for fear of being exposed to COVID-19.

Here, we used stochastic modelling of the fraction of COVID-19 cases captured at each step in the reporting chain to estimate the under-ascertainment of COVID-19 in Toronto. While this method, used for foodborne infections and influenza,^22–24^ yielded slightly higher estimates than those reported in a COVID-19 seroprevalence study for Ontario during the same timeframe,^30^ the differences were not significant. Interestingly, we observed that the ratio from the seroprevalence study was lower (4.7) at the starting period and higher (22.6) at the end and was vice versa for the model-based estimates. The increasing pattern from the seroprevalence estimates could be due to the varying antibody response in those infected individuals that allowed more serum residue detection rate at the later stage than the cases captured in the surveillance system. On the other hand, in our study, as time passed, test availability improved, and more people were able to get tested and counted in the reports, reducing the model-based multiplier estimates. Regardless, estimates from serologic surveys can be limited by selection bias in the screened population and the varying antibody response with lasting time and specimen type. Using multiple data sources would facilitate a better understanding of the burden of COVID-19 infection.

Our study is subject to four limitations. First, the population survey used to understand individuals’ care-seeking was web-based and self-selected. Specific factors that may have influenced survey participation include a lack of awareness due to limited survey promotion, distrust of the survey’s anonymity, and fear of declaring COVID-19 symptoms, and reservations about completing a web survey hosted outside of Canada.^39^ Participants, who might be different from those who chose not to participate or did not have access to the survey, could have altered the estimates in both directions. Second, we used symptomatic proportion (~95%) from the reported case data, which was higher than any of those reported by studies in the literature (range: 23%–80%) since those who got captured in a surveillance system are highly likely to be symptomatic individuals. Third, the proportion of those who sought care that got tested was not very accurate and most influential but should be improved. Lastly, our estimates might not reflect the situation in the entire city as Toronto’s population is not homogenous (e.g. the difference in socioeconomic status) vis-à-vis COVID-19 infection rate.^40^

Despite the limitations, our study employed a method that allowed us to account for our uncertainty about the actual values of the proportions reported at the steps in the reporting chain, using available data sources. Moreover, our approach enables local health units to identify the steps in the reporting chain at which cases are undercounted. This can be adapted to various contexts, including those related to new variants, added tools (e.g. rapid antigen tests), and even other changes to health-seeking behaviour.

## Conclusions

To conclude, during the first wave of the COVID-19 pandemic, only a fraction of the total COVID-19 cases occurring in the community was reported to Toronto Public Health, with an estimated 18 infections occurring in the community for each COVID-19 case reported to the local public health unit. As reported numbers do not reflect the actual infection rate in the community, policymakers, program planners, and local public health units should consider the ratio of reported versus potential missed cases in such infectious disease outbreaks.

## Supplemental Material

sj-docx-1-phj-10.1177_22799036231174133 – Supplemental material for Estimating the Under-ascertainment of COVID-19 cases in Toronto, Ontario, March to May 2020 Click here for additional data file. Supplemental material, sj-docx-1-phj-10.1177_22799036231174133 for Estimating the Under-ascertainment of COVID-19 cases in Toronto, Ontario, March to May 2020 by Binyam N Desta, Sylvia Ota, Effie Gournis, Sara M Pires, Amy L Greer, Warren Dodd and Shannon E Majowicz in Journal of Public Health Research
