# Supplementary material for: Estimating the Under-ascertainment of COVID-19 cases in Toronto, Ontario, March to May 2020
Source: J Public Health Res. 2023 May 12;12(2):22799036231174133. doi: 10.1177/22799036231174133 (PMC10184215; doi:10.1177/22799036231174133)
Supplement: sj-docx-1-phj-10.1177_22799036231174133 – Supplemental material for Estimating the Under-ascertainment of COVID-19 cases in Toronto, Ontario, March to May 2020 [file sj-docx-1-phj-10.1177_22799036231174133.docx]

**Estimating the Under-ascertainment** **of COVID-19 cases in Toronto, Ontario, March to May 2020**

Binyam N. Desta, Sylvia Ota, Effie Gournis, Sara M. Pires, Amy L. Greer, Warren Dodd, and Shannon E. Majowicz

**Supplemental Materials**

**Sensitivity Analysis** Page 2

**Supplemental Table S1** Page 3

**Additional Notes on Data Sources** Page 5

**Data Completeness by Variable – Population Survey** Page 6

**Supplemental Table S2** Page 6

**Data Matched with Testing Window – Three Data Sources** Page 7

**Supplemental Table S3** Page 7

**COVID-19 Asymptomatic Proportion Estimates in Other Similar Settings** Page 9

**Supplemental Table S4** Page 9

**Sensitivity Analysis**

We conducted a sensitivity analysis to assess the influence of our assumptions and analytic decisions on the under-ascertainment estimates from our main analysis via fourteen scenarios, by compared the percent change in the under-ascertainment multiplier under different scenarios. The impacts of the twelve modelling decisions/assumptions are shown in Table S1. Overall, the differences made by including scenarios listed in Table S1 were negligible. The first scenario that showed the highest deviation from our under-ascertainment mean estimate was for the proportion of those who sought care that got tested when using a beta distribution where the denominator and numerators values were derived solely from the population survey. The other scenario that showed the second-highest deviation from our under-ascertainment mean estimate was the proportion of those with COVID-19 in the community who were symptomatic, replaced with the smallest proportion (23%) from studies in the literature.

**Table S1.** Sensitivity analysis illustrating the estimated COVID-19 under-ascertainment multiplier in Toronto, Ontario, March to May, 2020, under different modeling decisions and assumptions

| Scenario | Multiplier for the Entire Time Period | Multiplier by Testing Window | | |
| --- | --- | --- | --- | --- |
|  |  | **1st** | **2nd** | **3rd** |
|  |  | **Up to March 12, 2020** | **March 13 to April 9, 2020** | **April 10 to May 23, 2020** |
| MAIN RESULTS, REPEATED FROM TABLE 2 | 18.395 | 28.001 | 19.846 | 13.879 |
| REPORTED CASE DATA | | | | |
| The number of cases reported determined using reported and specimen collection date combined | 18.359 | 23.675 | 17.193 | 13.926 |
| The number of cases reported determined using specimen collection date only | 18.547 | 23.907 | 17.450 | 14.635 |
| WEEKLY TESTING DATA | | | | |
| The two suppressed cell counts replaced as one and five and included in the number of positive tests | 18.443 | 30.114 | 19.835 | 13.889 |
| The two suppressed cell counts replaced as five and five and included in the number of positive tests | 18.345 | 31.305 | 19.766 | 13.836 |
| The two suppressed cell counts replaced as three and three and included in the number of positive tests | 18.443 | 30.114 | 19.835 | 13.889 |
| The two suppressed cell counts replaced as one and one and included in the number of positive tests | 18.477 | 28.670 | 19.875 | 13.870 |
| TEST SENSITIVITY | | | | |
| The minimum test sensitivity value replaced to be 38%, and included in the proportion of those who sought testing that tested positive | 24.394 | 37.495 | 26.353 | 18.454 |
| POPULATION SURVEY | | | | |
| For the proportion of those who sought care that got tested, using a beta distribution where the denominator and numerators were values derived from the population survey | 72.626 | 108.394 | 80.453 | 43.513 |
| For the proportion of those who sought care that got tested, replacing the maximum value as 90% for the pert distribution | 18.513 | 28.386 | 19.912 | 14.020 |
| For the proportion of those who sought care that got tested, replacing the maximum value as 95% for the pert distribution | 18.432 | 28.389 | 19.913 | 13.961 |
| Individuals who sought care via Ontario’s online COVID-19 self-assessment tool included in the number who sought testing | 10.476 | 16.687 | 11.653 | 7.764 |
| Individuals who fulfilled any of the criteria in the three windows included to the proportion of those with COVID symptoms who met the testing criteria, regardless of the time they met the criteria (Individuals with non-sensical dates, n=371) | 23.429 | N/A* | N/A | N/A |
| SYMPTOMATIC PROPORTION | | | | |
| For the proportion of those with COVID-19 in the community who were symptomatic, replacing to the smallest proportion (23%) from studies in the literature | 66.449 | 101.220 | 70.924 | 49.857 |
| For the proportion of those with COVID-19 in the community who were symptomatic, replacing to the smallest proportion (80%) from studies in the literature | 23.034 | 35.268 | 24.676 | 17.417 |

*N/A – Not Applicable

**Additional Notes on Data Sources**

Here we present additional details about the data manipulation process we undertook on the Toronto Public Health population survey data. Of the 1444 respondents, while the survey asked about symptoms since March 1, 2020, about 57 (3.9%) people reported onset dates before March 1. Four (2.8%) reported onset dates (one with no resolved date) from March 9 to April 03, 2001, where the reported symptoms include runny nose and sore throat, which are unlikely to last for many years; thus, we corrected the year to 2020 assuming it was a typo. We included 34 (2.4%) respondents who reported the onset date before March 1 since they reported the respective resolved date after March 1. We also included 8 (5.5%) respondents who reported an onset date only before March 1, assuming they could not report a resolved date if the symptoms were ongoing by the time they responded to the survey. We excluded nine (0.6%) people who reported both onset and resolved dates before March 1 and one with a resolved date only since they did not match the survey target (i.e., symptoms since March 1, 2020). We also excluded one respondent with an onset date only, which was after the end date (May 23, 2020) of our analysis window.

**Data Completeness by Variable – Population Survey**

**Table S2.** Data completeness by variable for the COVID-19 population survey (n=3,529), Toronto, Ontario, Canada, March to May, 2020

| Variable | Number missing (%) |
| --- | --- |
| Symptomatic Status | 1227 (34.77) |
| *Symptomatic respondents (n=1444)* | |
| Gender | 132 (9.14) |
| Age | 122 (8.45) |
| Onset Date | 361 (25.00) |
| Resolved Date | 817 (56.58) |
| Testing for COVID-19 | 317 (22.00) |
| Care seeking | 317 (22.00) |
| Chronic illnesses or underlying medical conditions | 317 (22.00) |

**Data Matched with Testing Window – Three Data Sources**

**Table S3.** Data matched with testing windows for the three data sets (COVID-19 population survey, weekly testing, and reported case data) in Toronto, Ontario, Canada, March to May, 2020

| Number of Responses / Data Points, by Data Source | Entire Time Period | By Testing Window | | |
| --- | --- | --- | --- | --- |
|  |  | **1st** | **2nd** | **3rd** |
|  |  | **Up to March 12, 2020** | **March 13 to April 9, 2020** | **April 10 to May 23, 2020** |
| REPORTED CASE DATA (n=5530 symptomatic and confirmed cases) * | | | | |
| TOTAL NO. REPORTED CASES (USING REPORTED DATE) INCLUDED IN THE ANALYSIS FOR EACH TIME PERIOD | 5530 | 57 | 1341 | 4132 |
| WEEKLY TESTING DATA** | | | | |
| For the 17 weeks falling completely within the testing window, the number positive | 6178 | 16 | 1215 | 4947 |
| For testing week March 8 to 14, the number positive divided proportionally by 5 days in the 1st window, 2 days in the 2nd window | 99 | 71 | 28 |  |
| For testing week April 5 to 11, the number positive divided proportionally by 5 days in the 2nd window, 2 days in the 3rd window | 652 |  | 466 | 186 |
| TOTAL NO. POSITIVES INCLUDED IN THE ANALYSIS FOR EACH TIME PERIOD | 6929 | 87 | 1709 | 5133 |
| POPULATION SURVEY DATA (n=1433 symptomatic respondents) *** | | | | |
| Those with both onset and resolved dates reported (606/1433; 42.29%) | | | | |
| Onset and resolved dates both falling within a single window | 340 | 28 | 279 | 33 |
| Onset date in the 1st, and resolved date in the 2nd, window | 154 | 154 | |  |
| Onset date in the 1st, and resolved date in the 3rd, window | 30 | 30 | | |
| Onset date in the 2nd, and resolved date in the 3rd, window | 81 |  | 81 | |
| Onset date in the 3rd, and resolved date after the 3rd, window | 1 |  |  | 1 |
| Those with onset dates only (456/1433; 31.82%) | 456 | 50 | 327 | 79 |
| TOTAL NO. SURVEY RESPONDENTS INCLUDED IN THE ANALYSIS FOR EACH TIME PERIOD | 1062 | 262 | 871 | 224 |

*****Span of reported date: January 23, 2020 - May 23, 2020.

******Start date of the first week: January 12, 2020; End date of the last week: May 23, 2020.

***Span of onset date: September 27, 2019 - May 30, 2020; Span of resolved date: January 2, 2020 - June 5, 2020.

**COVID-19 Asymptomatic Proportion Estimates in Other Similar Settings**

**Table S4.** Studies from high income countries on asymptomatic proportion of COVID-19 cases, January to November, 2020

| Author(s) & Year | Country | Study population (Population type, sampling, age) | Study period | Sample size (Tested) | Total number tested positive for COVID-19 (%) | Asymptomatic proportion (95% CI) |
| --- | --- | --- | --- | --- | --- | --- |
| Surveillance report | | | | | | |
| Spiterl et al., 2020  (1) | WHO European Region | Surveillance report from health facilities (general population – all ages) | January to February 2020 | NR | 31 | 0.065 (NR) |
| Toronto Public Health | Canada | All cases | January 1 – May 23, 2020 | NA | 10517 (NA) | 0.154 (NA) |
| Toronto Public Health | Canada | All cases | January 1, 2020 – July 26, 2021 | NA | 151243 (NA) | 0.174 (NA) |
| Toronto Public Health | Canada | Confirmed cases only | January 1 – May 23, 2020 | 9701 | 9701 (100) | 0.166 (NA) |
| Toronto Public Health | Canada | Confirmed cases only | January 1, 2020 – July 26, 2021 | 147155 | 147155 (100) | 0.175 (NA) |
| Toronto Public Health | Canada | Sporadic cases only | January 1 – May 23, 2020 | NA | 6025 (NA) | 0.081 (NA) |
| Toronto Public Health | Canada | Sporadic cases only | January 1, 2020 – July 26, 2021 | NA | 131277 (NA) | 0.150 (NA) |
| Toronto Public Health | Canada | Outbreak associated cases | January 1 – May 23, 2020 | NA | 4492 (NA) | 0.254 (NA) |
| Toronto Public Health | Canada | Outbreak associated cases | January 1, 2020 – July 26, 2021 | NA | 19966 (NA) | 0.174 (NA) |
| Screening in general population | | | | | | |
| Lavezzo et al, 2020  (2) | Italy | General population (Longitudinal study – No random sample) | February to March 2020 | 5155 | 102 (2.0) | 0.425 (0.315, 0.546) |
| Gudbjartsson et al., 2020  (3) | Iceland | General population (Random sampling) | March to April, 2020 | 13080 | 100 (0.8) | 0.430 (NR) |
| Chamie et al., 2020  (4) | USA | General population  (Longitudinal study – No random sample -≥4 years) | April, 2020 | 3871 | 83 (2.1) | 0.277 (NR) |
| Menachemi et al., 2020  (5) | USA | General population (Random sampling - ≥12 years) | April, 2020 | 3605 | 47 (1.7) | 0.442 (NR) |
| Snoeck et al., 2020  (6) | Luxemburg | General population  (Random sample) | April to May, 2020 | 1862 | 5 (0.3) | 0.200 (NR) |
| Petersen & Phillips, 2020  (7) | England | General population (Random sample) | April to June, 2020 | 36,061 | 115 (0.3) | 0.765 (0.677, 0.839) |
| Riley et al, 2020 (a, b, c)  (8) | England | General population (Random sample – 5 years and above) | October to November, 2020 | 932,072 | 3029 (0.3) | 0.470 (NR) |
| Screenings in defined population settings | | | | | | |
| Lombardi et al., 2020  (9) | Italy | Screening of defined population: Health care workers other than Nursing homes | February to March 2020 | 1573 | 138 (8.8) | 0.297 (NR) |
| Romao et al., 2020  (10) | Portugal | Screening of defined population: Health care workers | March, 2020 | 34 | 14 (41.2) | 0.210 (NR) |
| Treibel et al., 2020  (11) | UK | Screening of defined population: Health care workers | March to April, 2020 | 400 | 44 (11.0) | 0.270 (NR) |
| Ly et al., 2020  (12) | France | Screening of defined population: Residents and workers in different accommodation centers (shelters, hotels, and other residences) | March to April, 2020 | 1691 | 49 (7.0%) | 0.510 (NR) |
| Lan et al., 2020  (13) | USA | Screening of defined population: Grocery retail workers | May, 2020 | 104 | 21 (20.2) | 0.760 (NR) |

NA – Not applicable

NR – Not reported

**References**

1. Spiteri G, Fielding J, Diercke M, Campese C, Enouf V, Gaymard A, et al. First cases of coronavirus disease 2019 (COVID-19) in the WHO European Region, 24 January to 21 February 2020. Eurosurveillance [Internet]. 2020 Mar 5 [cited 2021 Aug 6];25(9):2000178. Available from: https://www.eurosurveillance.org/content/10.2807/1560-7917.ES.2020.25.9.2000178

2. Lavezzo E, Franchin E, Ciavarella C, Cuomo-Dannenburg G, Barzon L, Del Vecchio C, et al. Suppression of a SARS-CoV-2 outbreak in the Italian municipality of Vo’. Nat 2020 5847821 [Internet]. 2020 Jun 30 [cited 2021 Aug 6];584(7821):425–9. Available from: https://www-nature-com.proxy.lib.uwaterloo.ca/articles/s41586-020-2488-1

3. Gudbjartsson DF, Helgason A, Jonsson H, Magnusson OT, Melsted P, Norddahl GL, et al. Spread of SARS-CoV-2 in the Icelandic Population. N Engl J Med [Internet]. 2020 Jun 11 [cited 2021 Aug 6];382(24):2302–15. Available from: /pmc/articles/PMC7175425/

4. Chamie G, Marquez C, Crawford E, Peng J, Petersen M, Schwab D, et al. SARS-CoV-2 Community Transmission disproportionately affects Latinx population during Shelter-in-Place in San Francisco.

5. Menachemi N, Yiannoutsos CT, Dixon BE, Duszynski TJ, Fadel WF, Wools-Kaloustian KK, et al. Population Point Prevalence of SARS-CoV-2 Infection Based on a Statewide Random Sample — Indiana, April 25–29, 2020. Morb Mortal Wkly Rep [Internet]. 2020 Jul 24 [cited 2021 Aug 6];69(29):960. Available from: /pmc/articles/PMC7377824/

6. Snoeck CJ, Vaillant M, Abdelrahman T, Satagopam VP, Turner JD, Beaumont K, et al. Prevalence of SARS-CoV-2 infection in the Luxembourgish population-the CON-VINCE study. [cited 2021 Aug 16]; Available from: https://doi.org/10.1101/2020.05.11.20092916

7. Petersen I, Phillips A. <p>Three Quarters of People with SARS-CoV-2 Infection are Asymptomatic: Analysis of English Household Survey Data</p>. Clin Epidemiol [Internet]. 2020 Oct 8 [cited 2021 Sep 16];12:1039–43. Available from: https://www.dovepress.com/three-quarters-of-people-with-sars-cov-2-infection-are-asymptomatic-an-peer-reviewed-fulltext-article-CLEP

8. Riley S, Ainslie KEC, Eales O, Walters CE, Wang H, Atchison C, et al. REACT-1 round 6 updated report: high prevalence of SARS-CoV-2 swab positivity with reduced rate of growth in England at the start of November 2020.

9. Lombardi A, Consonni D, Carugno M, Bozzi G, Mangioni D, Muscatello A, et al. Characteristics of 1,573 healthcare workers who underwent nasopharyngeal swab for SARS-CoV-2 in Milano, Lombardy, Italy. medRxiv [Internet]. 2020 May 11 [cited 2021 Aug 6];2020.05.07.20094276. Available from: https://www.medrxiv.org/content/10.1101/2020.05.07.20094276v1

10. Romão VC, Oliveira-Ramos F, Cruz-Machado AR, Martins P, Barreira S, Silva-Dinis J, et al. A COVID-19 Outbreak in a Rheumatology Department Upon the Early Days of the Pandemic. Front Med. 2020 Sep 25;0:576.

11. Treibel TA, Manisty C, Burton M, McKnight Á, Lambourne J, Augusto JB, et al. COVID-19: PCR screening of asymptomatic health-care workers at London hospital [Internet]. Vol. 395, The Lancet. Elsevier; 2020 [cited 2021 Aug 6]. p. 1608–10. Available from: /pmc/articles/PMC7206444/

12. Ly TDA, Zanini D, Laforge V, Arlotto S, Gentile S, Mendizabal H, et al. Pattern of SARS-CoV-2 infection among dependant elderly residents living in long-term care facilities in Marseille, France, March–June 2020. Int J Antimicrob Agents [Internet]. 2020 Dec 1 [cited 2021 Sep 16];56(6):106219. Available from: /pmc/articles/PMC7661959/

13. Lan F-Y, Suharlim C, Kales SN, Yang J. Association between SARS-CoV-2 infection, exposure risk and mental health among a cohort of essential retail workers in the USA. Occup Environ Med [Internet]. 2021 Apr 1 [cited 2021 Sep 16];78(4):237–43. Available from: https://oem.bmj.com/content/78/4/237
